# Landscape, demographic, entomological, and climatic associations with human disease incidence of West Nile virus in the state of Iowa, USA

**DOI:** 10.1186/1476-072X-7-19

**Published:** 2008-05-01

**Authors:** John P DeGroote, Ramanathan Sugumaran, Sarah M Brend, Brad J Tucker, Lyric C Bartholomay

**Affiliations:** 1 GeoInformatics Training, Research, Education, and Extension Center, Geography Department, University of Northern Iowa, Cedar Falls, IA, USA; 2 Iowa Department of Public Health, Des Moines, IA, USA; 3 Department of Entomology, Iowa State University, Ames, IA, USA

## Abstract

**Background:**

West Nile virus (WNV) emerged as a threat to public and veterinary health in the Midwest United States in 2001 and continues to cause significant morbidity and mortality annually. To investigate biotic and abiotic factors associated with disease incidence, cases of reported human disease caused by West Nile virus (WNV) in the state of Iowa were aggregated by census block groups in Iowa for the years 2002–2006. Spatially explicit data on landscape, demographic, and climatic conditions were collated and analyzed by census block groups. Statistical tests of differences between means and distributions of landscape, demographic, and climatic variables for census block groups with and without WNV disease incidence were carried out. Entomological data from Iowa were considered at the state level to add context to the potential ecological events taking place.

**Results:**

Numerous statistically significant differences were shown in the means and distributions of various landscape and demographic variables for census block groups with and without WNV disease incidence. Census block groups with WNV disease incidence had significantly lower population densities than those without. Landscape variables showing differences included stream density, road density, land cover compositions, presence of irrigation, and presence of animal feeding operations. Statistically significant differences in the annual means of precipitations, dew point, and minimum temperature for both the year of WNV disease incidence and the prior year, were detected in at least one year of the analysis for each parameter. However, the differences were not consistent between years.

**Conclusion:**

The analysis of human WNV disease incidence by census block groups in Iowa demonstrated unique landscape, demographic, and climatic associations. Our results indicate that multiple ecological WNV transmission dynamics are most likely taking place in Iowa. In 2003 and 2006, drier conditions were associated with WNV disease incidence. In a significant novel finding, rural agricultural settings were shown to be strongly associated with human WNV disease incidence in Iowa.

## Background

West Nile virus was first recorded in the United States in 1999 and quickly spread across the country with 27,396 reported cases of WNV disease and 1025 fatalities through 2007 [1]. Some of the highest rates of WNV disease in the USA have occurred in the northern Midwest and plains states [1]. From 2002–2007, 23.6% of total WNV disease incidence occurred in Iowa, Minnesota, Nebraska, South Dakota, and North Dakota [1], while these states contain approximately 3.8% of the population of the continental USA. The disproportionate incidence in this area merits investigation distinct from the number of WNV related studies published about other areas of the country such as more densely populated Eastern states [2-6] and urban areas like Chicago and New York [7-9]. Few studies have described WNV landscape ecology/spatial epidemiology in northern Midwest and plains states [10,11].

West Nile virus is a vector-borne disease that is maintained in an enzootic cycle, transmitted primarily between avian hosts and mosquito vectors [12]. The spread and establishment of the virus throughout North America has been attributed to the large number of mosquito species that can be infected with the virus [13]. A number of mosquitoes have tested positive for the presence of WNV infection, but select *Culex *species are considered the most important vectors because of their inherent susceptibility that facilitates amplification and transmission of the virus. *Culex. pipiens *L. and *Cx. restuans *Theobald are thought to be the primary vectors in the north-eastern and north-central United States while *Cx. tarsalis *and *Cx. quinquefasciatus *are the primary vectors in much of the western United States [14]. As WNV moved westward in 2001–2004, the proportion of *Cx. tarsalis *positive mosquito pools out of total positive pools became much greater [15]. *Culex pipiens*, *Cx. restuans*, and *Cx. tarsalis *mosquitoes are all present in Iowa, but differ in habitat requirements. *Culex pipiens *is considered an urban and suburban mosquito species, *Cx. restuans *has been shown to be common in both rural and urban settings [16,17], and *Cx. tarsalis *is considered a primary rural WNV vector [18].

There have been a number of studies, at various scales, in which spatial and temporal data on climatic conditions, landscape, or demography were analyzed in relation to WNV dynamics in humans, mosquitoes, sentinels, wild birds, and horses.

### Demography

Demographic patterns can influence vector and host ecology and thus potential disease distribution. Ruiz et al. [19] broke Chicago and Detroit census tracts into groups based on land cover, elevation, housing, and demographic characteristics. They found that tracts classified as 'Inner Suburbs' had the highest number of human WNV disease cases in both cities. Gibbs et al. [20] found in Georgia that medium housing density in non-mountainous areas had higher probabilities of being associated with WNV positive birds and indicated this might be due to different vector habitat preferences.

### Landscape

Landscape attributes have an important effect on WNV disease dynamics and ecology by influencing host and vector presence, behaviour, and interactions. Ezenwa et al. [21] investigated the relationships between mosquito vectors, hosts (birds) and land cover in an area of Louisiana. They found that infection rates in *Culex *mosquitoes were negatively correlated with wetland area and that wetland area was positively correlated with non-amplifying bird hosts. In Mississippi, stream density, road density, slope, and Normalized Difference Vegetation Index (NDVI) data from satellite imagery were used along with seasonal climatic sub-models for predicting WNV disease incidence at a zip-code level. They found that 67% of human cases occurred in zip codes in the high risk category [22]. Vegetation indices, such as NDVI, derived from remote sensing, can be useful as they reflect vegetation vigour which in turn is influenced by landscape, temperature, and precipitation patterns.

### Climatic conditions

Climatic conditions can control timing and magnitude of vector populations, as well as influence host movement and behaviour. In the Red River Valley of North Dakota, Bell et al.[10] hypothesized that climatic conditions were poor (cool) in 2004 leading to low human WNV disease incidence. They also noted that 2003 (617 human cases) and 2005 (86 human cases) were similar in terms of climatic conditions. They hypothesized that the difference in cases was due to the immunity developed in passerine birds by 2005. However, in 2007, there were 367 cases reported in North Dakota [1]. In Wyoming, Zou et al. [23] used a degree day model to predict where and when the development of WNV reached a sufficient vector infectivity level. When compared to WNV disease incidence in humans, horses, and birds for 2003–2005 they predicted 91.3, 65.2, and 78.3% correctly at a county level. Landesman et al. [24] investigated data nationally at a county level and found that WNV disease incidence was most strongly associated with annual precipitation from the previous year. Shaman et al. [3] showed that WNV infection in sentinel chickens and human WNV disease incidence was associated with drought 2–6 months prior and modeled land surface wetting 0.5–1.5 months prior.

In this study, human WNV disease incidence data, at the census block group level in Iowa (2634 census block groups in Iowa with a mean population of 1111), were analyzed in relation to landscape, climatic, and entomological data to discern if unique landscape, climatic, and demographic associations with WNV exist. There have been few studies that have analyzed human WNV disease incidence data at spatial resolutions finer than the county scale, and none of these studies were conducted in rural areas of the Midwest [7,19,22]. Our analysis, at the scale of census block groups, allows for meaningful analysis of landscape and climatic variables. We carried out simple statistical analyses to discern important environmental associations with the occurrence of WNV disease in Iowa.

## Materials and methods

### Data Collection

Data on the date of each human WNV disease occurrence from 2002–2006 were attained at the census block group level from the Iowa Department of Public Health (IDPH). The IDPH policy for disclosure of reportable disease information was followed for the release of the human data. This policy indicates that reporting units must have a population of at least 300 before data can be released [25]. In this case, all census block groups with WNV disease incidence had a population of at least 500. Spatial data on 2000 census block group geography, land cover (2002), stream and road networks, digital elevation model (DEM), permitted irrigation points (>= 94.75 m^3^/day), and animal feeding operations were acquired from the Iowa Department of Natural Resources (IDNR) [26]. The census block group attribute data included a total population figure, which was used to calculate population density. The land cover data was produced by the IDNR from satellite imagery in 2002 and 2003 and has a resolution of 15 m. This dataset was generalized and reclassified from 17 to 9 classes (unclassified, water, wetlands, forest, grassland, agriculture/row crops, roads, commercial/industrial, residential). The 30 m resolution National Elevation Dataset DEM was used to produce a slope raster for Iowa. The topographic slope is considered, as flatter areas are more likely to foster areas of standing water important for mosquito development. Normalized Difference Vegetation Index (NDVI, 16 day composites, 250 m resolution) data from NASA's MODIS satellite were acquired for the years 2002–2005 from the University of Maryland Global Land Cover Facility (GLCF) [27]. The NDVI data were scaled to a range of 0–250 by the GLCF for easier display purposes. Raster surfaces, at a resolution of 4 km^2^, of annual minimum temperature, maximum temperature, precipitation, and dewpoint temperatures were acquired from the Oregon State University PRISM website [28]. The PRISM (Parameter-elevation Regressions on Independent Slopes Model) climate mapping system is a knowledge-based system that uses point measures of climatic data, digital elevation data and expert knowledge of complex climatic extremes to produce continuous climatic surfaces for the United States.

Data on mosquito abundance in Iowa were obtained from the Iowa-Mosquito.net website [29,30], that houses approximately 35 years of mosquito surveillance data collected using New Jersey Light Traps from throughout Iowa. *Culex *mosquito species abundances were compiled from the multiple collection locations in thirteen counties in Iowa for the years 2002–2006. Mosquito collections took place at 31–33 sites per year and with beginning dates from early May to late May and ending dates ranging from mid-September to early October. A more detailed description of the trapping regime in Iowa can be found in Socaet et al. [30]. Due to the difficulty in morphologically distinguishing adult *Cx. pipiens *and *Cx. restuans *individuals, these mosquitoes were grouped and are referred to as the *Cx. pipiens *group. Data on WNV infections in mosquitoes are presented based on previously unpublished data collected by the Iowa State University Medical Entomology Laboratory. From 2002–2006, multiple mosquito collection trips were made during peak vector activity periods. Mosquitoes were collected at multiple sites across the state using CO2 baited CDC and Mosquito Magnet^® ^traps or grass infusion baited gravid traps. Mosquitoes were transported on dry ice to the Medical Entomology Laboratory at Iowa State University (ISU) to be identified and pooled for virus isolation.

### Data Processing and Analysis

All human WNV disease cases from 2002–2006 in Iowa were geocoded using ESRI's ArcGIS software and a custom address locator, which included zip codes, by the IDPH. Approximately 79% of the 298 locations were geocoded successfully originally. All of the unsuccessful addresses were corrected and successfully geocoded by checking each mismatch in the recorded address and those in the address locator. The address points were then given the value of the census block group they fell in using a spatial join. The attribute table of the geocoded points was exported to a stand-alone table and all sensitive patient information was removed, so the only remaining information in the table was the week of year for each occurrence and the census block group identifier. Thus, the researchers outside of the IDPH were never given any human patient information.

All spatial processing was carried out using ArcGIS 9.2 and extensions of ArcGIS. The percentage of area falling in each individual land cover class within each census block groups was calculated using the 'Thematic Raster Summary' tool in the Hawth's Tools extension [31]. The density of streams and roads falling in each census block group was calculated by carrying out an intersection and then dividing the length of the roads or streams in each census block group by the area of that census block group. The number of irrigation points and animal feeding operations falling in each census block group was calculated by running the 'Count Points in Polygon' tool in the Hawth's Tools extension. Only more rural (defined as a population density of less than 0.5 persons/ha) census block groups were analyzed in order to distinguish whether the presence of irrigation and animal feeding operations might be important for the occurrence of WNV disease. Zonal statistics operations were carried out to calculate the mean elevation and mean slope, as well as the mean annual minimum temperature, maximum temperature, precipitation, and dewpoint temperature for each census block group. Iowa census block groups were separated into eastern and western regions by manually splitting the state approximately in half for comparing regional differences in some climatic and landscape associations. The average NDVI score for each year (March-September) was calculated for the state of Iowa. This time period was chosen as it was thought to encompass the potential WNV development season in Iowa. In addition, the mean NDVI score (March-September) by census block group for each of the years was calculated.

A Global Moran's I spatial autocorrelation test was carried out for the WNV disease incidence rate by census block group using distance threshold bands of 0, 2.5, 5, 10, and 20 kilometers. In addition, a hot-spot analysis was carried out based on the Getis-Ord Gi* statistic for distance thresholds of 2.5, 5, 10, and 20 kilometers. Both of these analyses were carried out using ArcGIS's Spatial Statistics tools [32].

Statistical analyses were carried out using SPSS 14.0 for Windows. T-tests in SPSS were used to test whether mean landscape, demographic, and climatic values for census block group with and without human WNV disease incidence were significantly different. When the Levene test showed a violation of the assumption of homogeneity of variance, the 'equal variances not assumed' option is reported [33]. For some of the analyses, including that of irrigation and animal feeding operations points, there were violations of the assumptions of normality, and thus the nonparametric Mann Whitney U test was used to test differences in distributions between census block groups with and without WNV disease.

## Results

The number of human WNV disease cases and the mean and median week of occurrence by year are shown in Table 1. For each year the greater percentage of WNV disease cases were in the western part of the state. The earliest median week of occurrence was in 2006 in week 33 and the latest was in 2002 in week 37. In eastern Iowa WNV disease cases occurred slightly later in the year (average week of occurrence = 35.22) than in western Iowa (34.93).

**Table 1:** West Nile virus cases by year and region.

**Year**	**Total Cases**	**Eastern Cases (%)**	**Western Cases (%)**	**Mean Week**	**Median Week**
**2002**	55	25 (45.5)	30(54.5)	37.18	37
**2003**	147	32 (21.8)	115 (78.2)	35.02	35
**2004**	23	11 (47.8)	12 (52.2)	33.83	35
**2005**	37	6 (16.2)	31 (83.8)	35.38	36
**2006**	36	11 (30.6)	25 (69.4)	32.08	33

### Cluster and Hot Spot Analysis

**Figure 1:**
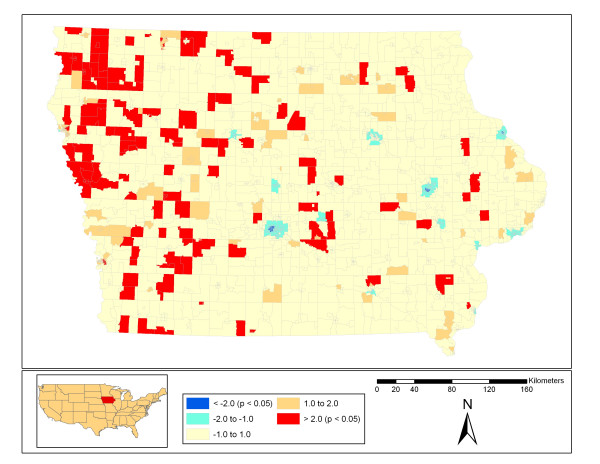
**Hot-spot analysis result at five km distance threshold**. Results of the hot-spot analysis (Getis-Ord Gi*) for human WNV disease rate from 2002–2006 by census block group with a 5 km threshold analysis distance. The values above 2.0 represent statistically significant hot-spots while below -2.0 are statistically significant cold-spots. The majority of census block groups fall in the statistically insignificant middle categories.

Tests showed that there was statistically significant (p < 0.05) spatial autocorrelation in WNV disease incidence rates at all distance thresholds tested. The results of spatial autocorrelation analysis for the different distance thresholds are as follows: distance threshold = 0 m, Moran's Index = 0.026, Z-score = 12.89; distance threshold = 2500 m, Moran's Index = 0.034, Z-score = 2.87; distance threshold = 5000 m, Moran's Index = 0.034, Z-score = 4.33; distance threshold = 10000 m, Moran's Index = 0.036, Z-score = 6.23; distance threshold = 20000 m, Moran's Index = 0.038, Z-score = 7.71. Each of the hot-spot analyses for human WNV disease rates (with differing distance thresholds) showed statistically significant hot-spots mainly in the western half of Iowa with significant cold-spots in urban areas. Figure 1 shows the results for the five km hot-spot analysis. The red areas indicate statistically significant hot-spots while the dark blue represent statistically significant cold spots for human WNV disease rates. The other threshold distances showed similar patterns with larger areas being statistically significantly hot or cold at the 20 km distance.

### Demographic, landscape, and climatic analysis

#### Demography

**Figure 2:**
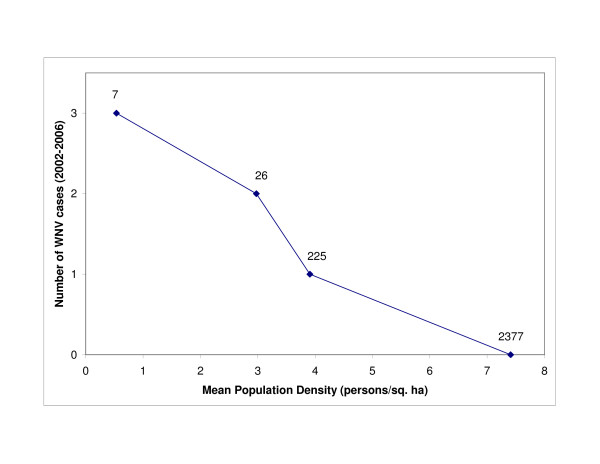
**Mean population density in census block groups with zero to three WNV human disease cases (2002–2006)**. On the x-axis is the mean population density (persons/ha^2^) for census block groups calculated according to the number of WNV disease cases (i.e. 0, 1, 2, or 3 cases). The number of census blocks which had that number of cases is shown above the point in the graph.

**Figure 3:**
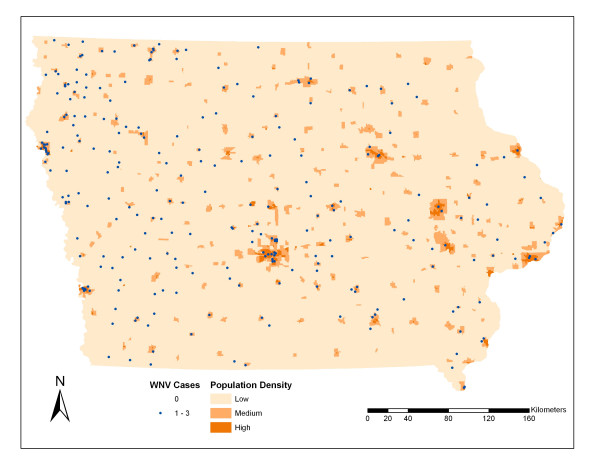
**Centroids of census block groups with WNV disease overlain on Iowa population density**. The centroids of those census block groups with WNV disease are overlain on a layer of populations density spit into three classes (Low = 0 – 0.5 persons/ha, Medium = 0.5 – 4 persons/ha, and High > 4 person/ha).

There was a statistically significant difference (t-value = 5.65, df = 2633, *P = *1.7E-8) in mean population density between census block groups with (N = 258, mean = 3.72, SD = 7.09) and without WNV disease incidence (N = 2377, mean = 7.41, SD = 10.2). Figure 2 shows the mean population density of census block groups with 0–3 incidences of human WNV disease from the period 2002–2006. Figure 3 illustrates the distribution of census block group centroids which had WNV disease cases overlain on a map of population density. There was not a statistically significant difference (t-value = -1.43, df = 256, *P *= 0.15) between eastern (mean = 2.77) and western (mean = 4.14) population densities in census block groups with WNV disease incidence.

#### Landscape

**Figure 4:**
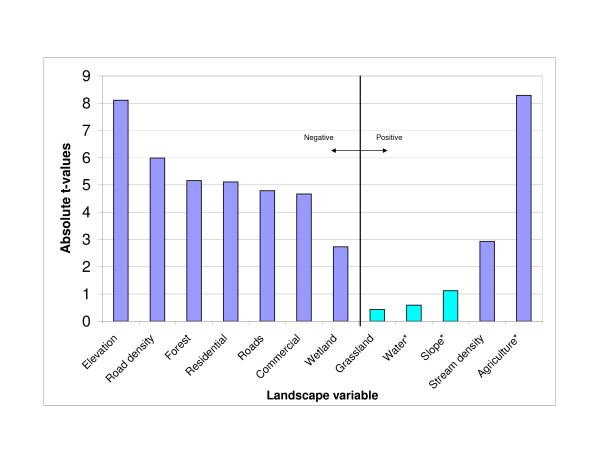
**Absolute t-values for landscape variables based on Students t-test**. This graphic represents the absolute t-value from the Students t-test comparing the means of landscape variables for census block groups with and without human WNV disease incidence. Those landscape variables to the left of the vertical line are ones in which there were lower means for census block groups with WNV disease incidence (negative) and those to the right were ones in which there were higher means for census block groups with WNV disease incidence (positive). All except grassland, water, and slope were significant (*P *<= 0.006). Those labelled with * used the 'equal variance not assumed' version of the t-test in SPSS. The agriculture class was reclassified from alfalfa, corn, soybeans, and other row crops.

**Figure 5:**
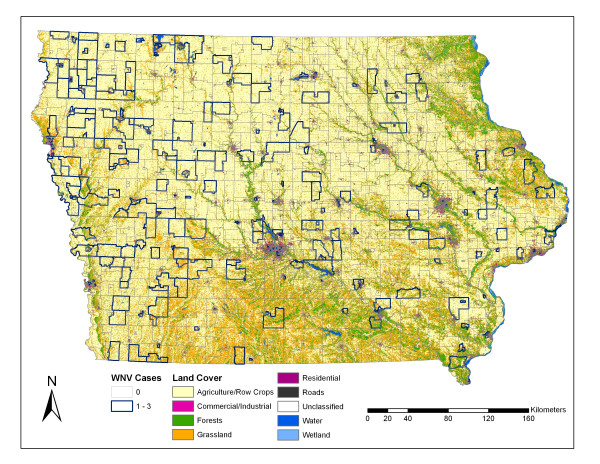
**Census block groups with and without WNV disease overlain on Iowa 2002 land cover**. The census block groups with WNV disease are highlighted with a darker outline. The land cover dataset was produced by the Iowa Department of Natural Resources and metadata can be found at [43].

**Figure 6:**
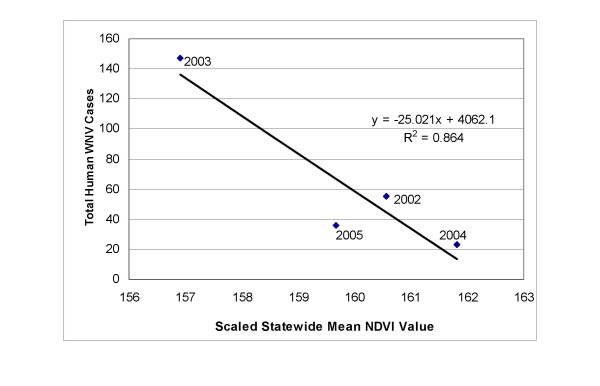
**Human WNV disease cases in Iowa (2002–2005) compared to state mean NDVI scores**. Total human WNV disease cases in Iowa by year (2002–2005) plotted against scaled mean NDVI scores from 2002–2005. The mean NDVI scores were calculated by calculating an average NDVI raster based on 14 separate scenes from March until September of each year. The mean NDVI score for each year was then calculated from this raster and plotted against the number of WNV disease cases.

The absolute t-values resulting from the T-tests comparing landscape variables in census block groups with or without WNV disease incidence is presented in Figure 4. Figure 5 represents the land cover of Iowa with census block groups overlain. Statistically significant higher mean stream density and proportion of agricultural areas were found in census block groups with human WNV disease incidence. Statistically significant lower mean road density and proportional area of wetlands, forests, commercial land use, and residential land use were found in census block groups with human WNV disease incidence. The Mann Whitney U test comparing the mean number of animal feeding operations and irrigation points in rural census block groups (N = 955), with and without human WNV disease incidence, showed statistically significant differences between the sample distributions indicated by the mean rank. For animal feeding operations, those census block groups with WNV disease incidence (N = 138, 161 WNV disease cases) had a higher mean rank (581.3) than those census block groups without WNV disease incidence (N = 817, mean rank = 460.55) (Z = -4.822, *P *= 1.42E-6). For irrigation points, those census block groups with WNV disease incidence (N = 138) had a higher mean rank (522.54) than those census block groups without WNV disease incidence (N = 817, mean rank = 470.48) (Z = -2.615, *P *= 0.009). There was a negative relationship at the state level between the mean NDVI and the number of human WNV disease cases (Figure 6) for the years 2002–2005. When the mean NDVI by census block groups was analyzed, there were no statistically significant differences in any of the years between those with or without human WNV disease incidence.

#### Climatic conditions

The test of means for climatic variables in census block groups with and without human WNV disease incidence for year of occurrence is presented in Table 2. In 2003 there was significantly (*P *< 0.01) less precipitation, lower mean dewpoint temperatures, and lower minimum temperatures in census block groups that had human WNV disease occurrence. In 2004 and 2005, there were significantly (*P *<= 0.02) lower minimum temperatures in census block groups with human WNV disease incidence. In 2006, there was significantly (*P *= 0.006) lower precipitation in census block groups with WNV disease incidence. The test of means for climatic variables in census block groups with and without human WNV disease incidence for preceding year is presented in Table 3. In the years 2003, 2004, and 2005 there were significantly lower precipitation, dewpoint temperatures, and minimum temperatures in the preceding year in census block groups with WNV disease incidence.

**Table 2:** T-test statistics for climatic variables for year of occurrence. The table shows the t-test statistics for climatic variables (in year of WNV disease occurrence) in census blocks with and without WNV disease incidence. All of the t-tests used 'equal variance not assumed' version of test in SPSS.

**Variable**	**Year**	**Degrees of Freedom**	**With WNV Mean**	**Without WNV Mean**	**t-value**	***P***
**Precipitation**	2002	54	768	763	-0.33	0.74
	2003	140	719	762	-5.63	**<0.001**
	2004	22	848	850	0.12	0.901
	2005	35	709	698	0.66	0.515
	2006	35	743	779	-2.9	**0.006**
**Dewpoint**	2002	54	4.35	4.4	0.50	0.62
	2003	140	3.16	3.42	-3.38	**<0.001**
	2004	22	4.61	4.33	-1.71	0.1
	2005	35	4.73	4.74	-0.10	0.922
	2006	35	4.86	5.01	-1.09	0.283
**Max. Temp**.	2002	54	15.7	15.7	-0.37	0.71
	2003	140	15.2	15.1	0.70	0.483
	2004	22	15.2	14.8	-1.86	0.076
	2005	35	15.7	15.8	-0.55	0.583
	2006	35	16.2	16.1	0.28	0.783
**Min. Temp**.	2002	54	3.71	3.82	0.66	0.52
	2003	143	2.73	3.09	-3.75	**<0.001**
	2004	23	3.17	3.68	-2.51	**0.02**
	2005	35	3.65	4.04	-2.74	**0.01**
	2006	35	4.29	4.55	-1.56	0.128

**Table 3:** T-test statistics for climatic variables for year preceding occurrence. The table shows the t-test statistics for climatic variables (from year preceding WNV disease occurrence) in census blocks with and without WNV disease incidence. All of the t-tests used 'equal variance not assumed' version of test in SPSS.

**Variable**	**WNV Occurrence Year**	**Degrees of Freedom**	**With WNV Mean**	**Without WNV Mean**	**t-value**	***P***
**Precipitation**	2002	54	887	879	0.56	0.58
	2003	145	693	767	-8.19	**<0.001**
	2004	23	702	761	-4.27	**<0.001**
	2005	35	819	848	-2.13	**0.041**
	2006	35	712	698	0.74	0.464
**Dewpoint**	2002	54	4.83	4.87	-0.37	0.71
	2003	140	3.97	4.43	-6.01	**<0.001**
	2004	22	3.04	3.41	-2.22	**0.037**
	2005	35	4.29	4.61	-2.36	**0.024**
	2006	35	4.71	4.74	-0.23	0.818
**Max. Temp**.	2002	54	15.4	15.3	-0.57	0.57
	2003	140	15.7	15.7	0.31	0.76
	2004	22	14.8	15.1	-1.88	0.074
	2005	35	15.1	15.2	-0.68	0.5
	2006	35	15.8	15.8	0.07	0.94
**Min. Temp**.	2002	54	3.93	4.02	-0.57	0.57
	2003	143	3.32	3.84	-5.48	**<0.001**
	2004	23	2.58	3.07	-2.47	**0.021**
	2005	35	3.18	3.68	-2.97	**0.005**
	2006	35	3.77	4.04	-1.48	0.147

In some instances, there were differences in climatic associations with WNV disease cases in the eastern and western regions of Iowa, as presented in Table 4. In 2002, in the west, and in 2006 in the east, there were statistically significant differences between precipitation in census block groups with (more precipitation) and without WNV disease incidence, while in 2003 there was significantly less precipitation in census blocks with WNV disease incidence in the western region of the state. In each year there were lower average minimum temperatures in census block groups with WNV disease incidence than those without in the western region of the state. These differences were statistically significant in all years except 2002. In 2003 and 2006 there were statistically significantly lower dewpoint temperatures in census block groups with WNV disease incidence in the western part of the state.

**Table 4:** Mann Whitney U test statistics for climatic variables broken by eastern and western regions. The table shows the Mann Whitney U test statistics for climatic variables in census block groups with and without WNV disease incidence for the year of occurrence broken into eastern and western regions. There were 24, 30, 11, 6, and 11 census block groups with WNV disease in the Eastern region in 2002, 2003, 2004, 2005, and 2006 respectively. There were 29, 99, 12, 29, and 24 census block groups with WNV disease in the Western region in 2002, 2003, 2004, 2005, and 2006 respectively.

**Variable**	**Year/Region**	**With WNV Mean Rank**	**Without WNV Mean Rank**	**z-score**	***P***
**Precipitation**	2002/East	701	695	-0.16	0.88
	2002/West	776	619	-2.31	**0.021**
	2003/East	733	694	-0.53	0.6
	2003/West	465	637	-4.57	**<0.001**
	2004/East	678	695	-0.14	0.89
	2004/West	626	623	-0.23	0.98
	2005/East	679	695	-0.96	0.92
	2005/West	623	624	-0.01	0.99
	2006/East	421	697	-2.27	**0.023**
	2006/West	526	625	-1.35	0.18
**Dewpoint**	2002/East	708	695	-0.74	0.94
	2002/West	581	625	-0.65	0.52
	2003/East	747	694	-0.71	0.48
	2003/West	479	636	-4.15	**<0.001**
	2004/East	516	696	-1.49	0.14
	2004/West	512	625	-1.08	0.28
	2005/East	547	695	-0.91	0.37
	2005/West	616	624	-0.11	0.91
	2006/East	881	694	-1.55	0.12
	2006/West	450	627	-2.4	**0.017**
**Max. Temp**.	2002/East	780	694	-1.04	0.3
	2002/West	579	625	-0.67	0.5
	2003/East	730	695	-0.49	0.63
	2003/West	607	636	-0.48	0.64
	2004/East	536	696	-1.32	0.19
	2004/West	412	626	-2.05	**0.041**
	2005/East	532	696	-1.0	0.32
	2005/West	557	625	-1.01	0.31
	2006/East	877	694	-1.51	0.13
	2006/West	559	625	-0.88	0.38
**Min. Temp**.	2002/East	741	694	-0.57	0.57
	2002/West	532	626	-1.38	0.17
	2003/East	712	695	-0.234	0.81
	2003/West	483	636	-4.04	**<0.001**
	2004/East	598	695	-0.80	0.42
	2004/West	397	626	-2.19	**0.029**
	2005/East	556	696	-0.85	0.40
	2005/West	486	627	-2.08	**0.037**
	2006/East	820	694	-1.04	0.30
	2006/West	397	628	-3.12	**0.002**

### Entomological data

**Figure 7:**
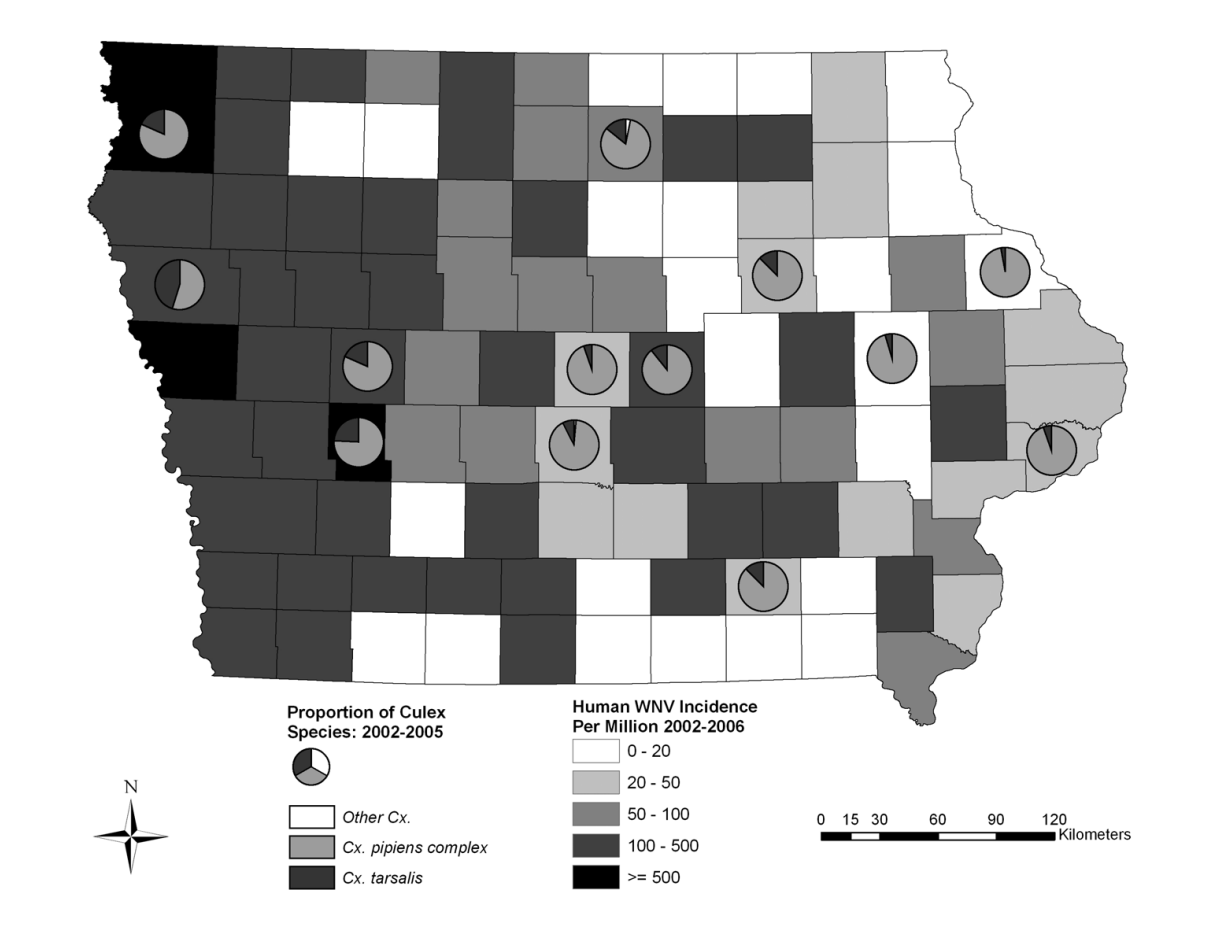
**The proportion of *Culex *mosquitoes and the WNV disease incidence rate in Iowa counties**. The mosquito proportions were derived from the databases at the Iowa State University Medical Entomology Laboratory IowaMosquito.net website while the WNV disease incidence rates were derived by dividing the number of cases in a county by the population and multiplying by one million to get the rate per million people.

In Iowa, there is a spatial shift in the distribution of key vector species in the genus *Culex *as measured by mosquito collections in New Jersey Light Traps (NJLT) [30]. Throughout the state, the *Cx. pipien *group are the most common species collected but *Cx. tarsalis *proportions go up in the western part of the state (Figure 7). Table 5 shows the timing of human WNV cases and timing and total number of *Cx. pipiens *group and *Cx. tarsalis *by week as collected in NJLT traps, as well as when positive pools of these mosquito species were found from 2002–2006. The mosquitoes both had similar temporal occurrences, with the highest percentage of mosquitoes in an individual week caught in week 26. There was a slight dip in *Cx. tarsalis *collections in weeks 28–30, and then a rebound with approximately 33% collected from weeks 31–34. From 2002–2006 there were 57 WNV positive mosquito pools found throughout the state, out of 3240 pools tested. The *Cx. pipiens *group accounted for 41 of the positive pools with the rest being *Cx. tarsalis *(13), *Cx. erraticus *(2), and *Aedes trivittatus *(1) Out of the 13 positive WNV pools of *Cx. tarsalis *mosquitoes found, 12 were in the western half of the state. However, positive pools of the *Cx. pipiens *group were found throughout the state. Overall, 64.5% of positive WNV mosquito pools were found in the western part of the state. The majority of WNV cases (62.8%) and positive mosquito pools (*Cx. tarsalis *= 77%, *Cx. pipiens *group = 56.2%) were found in weeks 34–38. Positive *Cx. pipiens *group pools were found both earlier and later than *Cx. tarsalis *pools.

**Table 5:** Timing of WNV cases, *Culex *mosquito collections, and positive *Culex *pools from 2002–2006 in Iowa. Epidemiologic and entomologic data were compiled for the years 2002–2006 and are presented according to the ISO week of the year. Human case events are presented as reported by the Iowa Department of Public Health. Mosquito populations are derived using population data generated by [44]. Populations are expressed as a % of the total mosquitoes collected for each respective species. Positive WNV pools are expressed as a % of the total positive pools listed in the summation.

Week of the Year	% Human WNV Cases	% Population as Measured with NJTL's		% WNV Positive Pools	
		*Cx. tarsalis*	*Cx. pipiens *group	*Cx. tarsalis*	*Cx. pipiens *group

19	0.3	0.0	0.0		
20	0.0	0.0	0.0		
21	0.0	0.0	0.6	0.0	0.0
22	0.0	1.6	3.3	0.0	0.0
23	0.3	5.8	6.1	0.0	0.0
24	0.0	4.0	7.9	0.0	4.9
25	0.3	5.3	10.1	0.0	0.0
26	1.0	**11.5**	**10.2**	0.0	2.4
27	0.7	9.3	7.8	0.0	0.0
28	2.3	5.7	4.0	0.0	0.0
29	0.7	5.0	5.2	7.7	2.4
30	3.0	6.6	5.5	0.0	0.0
31	4.0	10.2	5.9	0.0	2.4
32	5.7	7.5	5.7	0.0	4.9
33	8.7	7.8	4.8	15.4	**22.0**
34	8.1	7.3	5.6	0.0	4.9
35	14.4	3.2	4.3	**46.2**	9.8
36	**16.8**	2.9	5.1	7.7	12.2
37	16.1	2.5	4.5	23.1	17.1
38	7.4	2.6	3.0	0.0	12.2
39	5.0	0.9	0.4	0.0	4.9
40	2.0	0.0	0.0	0.0	0.0
41	1.3	0.0	0.0	0.0	0.0
42	0.7	0.0	0.0	0.0	0.0
43	0.7	0.0	0.0	0.0	0.0
44	0.0	0.0	0.0	0.0	0.0
45	0.0	0.0	0.0	0.0	0.0
46	0.3	0.0	0.0	0.0	0.0
Total	298 total cases	6086	33255	13(+)/193 pools	41(+)/788 pools

## Discussion

Human WNV disease incidence first occurred in significant numbers in Iowa in 2002 and peaked in 2003 with 147 cases. This is consistent with the second year of significant intrusion having the highest number of human cases. Illinois (884), Michigan (614) and Ohio (441) had the highest number of human cases in 2002, while in 2003 Colorado (2947), Nebraska (1036), and South Dakota (1039) had the highest number of human cases in the country. In each of these states, the virus was first detected in a significant amount in the previous year [1]. There are many possible reasons for these reductions after the peak year. These reasons could include public awareness, increased mosquito abatement, and a build up of immunity in bird and mammal populations [10]. Immune birds are considered dead-end hosts like humans [5]. Although none of these states has matched the peak number of cases, there have been a large number of cases since 2004 [1].

Human cases of West Nile virus have occurred throughout Iowa, with a greater concentration in the western part of the state (Figures 1 and 5). The analysis of spatial autocorrelation for human WNV disease rates showed significant hot-spots in Western Iowa over the course of the study period and significant cool spots in some urban areas. States directly to the west of Iowa, including Nebraska (10.9% of cases, 0.6% of population in continental USA) and South Dakota (6.2% of cases, 0.26% of population), have had some of the highest WNV disease rates in the country [1,34]. In Iowa, using the east-west split as described earlier, Eastern Iowa has 0.54% of the continental USA population with 0.36% of the WNV disease cases from 2002–2006, while Western Iowa has 0.45% of the continental USA population with 0.9% of the WNV human disease cases [1]. This higher incidence rate and the increasing proportion of *Cx. tarsalis *trapped in the western half of the state suggest that the western half of Iowa exhibits vector-host ecology more similar to that in the states to the west [10,35]. *Culex tarsalis *mosquitoes feed on both mammals and birds and thus are more likely to serve as a bridge vector to humans than mosquitoes of the *Cx. pipiens *group [16].

### Demography

In a novel finding, it was shown that human cases of WNV disease occurred in more rural settings in Iowa. This is opposed to eastern studies which showed more urban/suburban occurrence [7,19,20]. Figure 2 demonstrates that census block groups with an increasing number of human WNV disease cases (zero to three cases) had progressively lower population densities, while Figure 3 also demonstrates the rural nature of cases. There have been a limited number of studies that specifically investigated population density [7,11,19,20] in relation to WNV disease incidence. In Chicago and Detroit, areas classified as 'Inner Suburbs' with moderate population density had the highest percentage of WNV disease cases [7,19]. Similarly in Georgia, moderate housing density was associated with WNV disease incidence [20]. In Nebraska, the highest seroprevalence of WNV, estimated by blood donor data, was in the most sparsely populated part (western) of the state [11]. In Iowa, the western half of the state has a lower population density (0.17 persons/ha) than the eastern half (0.23 persons/ha). However, the census block groups with WNV disease incidence had a lower population density (although not a statistically significant difference) in the eastern part of the state. There were 213 (71.5%) cases in the western part of the state. The nine most densely populated census block groups with WNV disease incidence are in the western part of the state. It is possible this was caused by a higher overall WNV infection rate in mosquitoes in the Western part of the state. This is supported by the fact more positive WNV mosquito pools were found in the western half (64.5%) of the state. Figure 1 demonstrates that several of the cities in Eastern Iowa show up as being cold spots for human WNV disease rates, while none of the western cities do. In Sioux City, which is along the Missouri River on the western border, there were 13 cases in densely populated census block groups. Rural census block groups around this city also had WNV disease incidence. It is possible the movement of vectors or hosts between the rural-urban interface might have led to the urban WNV disease cases. Also, the fact that in the western part of the state both *Cx. pipiens *group and *Cx. tarsalis *positive mosquito pools were found extensively, could help explain both the urban and rural human disease occurrence. It must be noted that due to logistical constraints of running a continuous collection regime, the NJLT locations are often located in or near towns or cities and thus sampling of rural areas is likely a bit under-represented. This could lead to an under representation of *Cx. tarsalis *mosquito populations and positive pools, as they are a very rural mosquito.

### Landscape

The results of the land cover and land use analysis supported the rural nature of WNV disease incidence in Iowa. The most significant difference in land cover mean proportions between census block groups with and without WNV disease was for agricultural/row crop areas (Figure 4). Figure 5 demonstrates the large number of agriculturally dominated census block groups with WNV disease occurrence. In addition, the land use indicators of permitted agricultural irrigation and animal feeding operations were associated with the census block groups with WNV disease incidence. In California, Reisen et al. [35] suggested that farmhouse environs provide 'islands' of elevated vegetation used by birds for nesting and by *Cx. tarsalis *for host-seeking and resting. This could help explain the agricultural association in Iowa also. Total crop sales were shown to be a significant independent predictor of WNV disease incidence by county in Colorado, Nebraska, Louisiana, and Pennsylvania in 2002–2003 [36]. However, the number of irrigated acres was not a significant predictor in that study. In the Great Plains region [37], and in California [38], large populations of *Cx. tarsalis *were associated with irrigated areas. In 2003, the year of highest WNV disease incidence in Iowa, census block groups with WNV disease incidence had significantly less precipitation than those without. In the drier years of 2003 and 2005, a higher proportion of census block groups with WNV disease incidence were ones which contained permitted irrigation points compared to the other four years. The interaction of agricultural activity, such as irrigation and intensive animal operations in relation to mosquito abundance, and WNV disease incidence needs to be studied more thoroughly in Iowa. Census block groups having WNV disease incidence encompassed less forested areas than those without. Many heavily forested areas have low population densities. *Culex tarsalis *larval habitats include newly-created sunlit surface pools surrounded by grasses [39] and thus forested habitat likely does not support *Cx. tarsalis *larvae. Also, *Cx. pipiens *is considered an urban mosquito and thus also might not be prevalent in or near forested areas. If *Cx. tarsalis *is the primary vector in Iowa, then we might expect there to be higher proportion of grasslands associated with WNV disease, but there was no significant difference in grassland proportions in census block groups with and without WNV disease. Higher proportions of the built environment land cover classes (roads, commercial, residential) were associated with those census block groups without WNV disease. This implies that the urban-centric *Cx. pipiens *species might play a more minor role in Iowa as compared to the rural *Cx. tarsalis *species. Urban and suburban land use was an important predictive variable for WNV disease incidence in Georgia [20] and Illinois [19] where the more urban *Cx. quinquefasciatus *and *Cx. pipiens *are considered the primary vectors respectively. There is not a large amount of wetland area in Iowa (~0.5% of state), and it is often in quite rural areas, but it was still somewhat surprising that wetlands were shown to be less common in census block groups with WNV disease. This could be due to host-vector interactions that are not well understood. In Louisiana, Ezenwa et al. [21] found that WNV infection rates in *Culex *mosquitoes declined with increasing wetland cover. They found that the area of wetlands was significantly and negatively correlated with the passerine to non-passerine bird ratio and they theorize that the non-passerine bird species act as dilution hosts. At a state level there was a negative relationship between NDVI score and WNV disease cases as indicated in Figure 6. This is interesting, in that warmer and drier years (2003 and 2002) had more WNV disease than a cooler and wetter year (2004). Given that this was a March-September NDVI average, and for only a four year period, limited conclusions can be made.

### Climate

Some interesting year-by-year associations were made between climatic data and WNV disease incidence. However, analysis of climatic data from the year of WNV disease observations did not reveal any consistent trends. In 2003, census block groups with WNV disease had significantly less precipitation, lower dewpoint and average annual minimum temperatures. These relationships were very strong in the western half of the state but reversed in the eastern part of the state indicating varying disease dynamics. In 2003, there were relatively wet conditions in May, June, and July, but then a very dry August. The mean and median week of WNV disease occurrence was at the end of August. This is opposite to the pattern described by Shaman et al. [3], where early season drought followed by later season rainfall led to large outbreaks. The mean annual minimum temperature was always lower in census block groups with WNV disease incidence and significantly so in 2003, 2004, and 2005. This relationship was true for all years in the western part of the state with 2003, 2004, 2005, and 2006 having significantly lower average minimum temperatures in census block groups with WNV disease incidence. This is a bit contradictory to other studies that conclude higher temperatures could potentially increase probability of WNV transmission [23,40]. The magnitude in differences on an annual basis (e.g. approximately 0.5°C) are not huge and it is unclear in what way this would have an effect on WNV disease incidence. However, given that we considered only annual average climatic variables, it is possible that more detailed temporal data might reveal different associations. Temperatures are generally lower in the northern part of the state, which also has significant agricultural areas and animal feeding operations. The influence of temperature needs to be examined more thoroughly to help understand its effect on human WNV disease incidence in Iowa. The differences between eastern and western halves of the state suggest that different vector-host-disease dynamics have been occurring in different parts of the state. A possible difference is that drier conditions in western Iowa lead to higher levels of irrigation which in turn provides good habitat for *Cx. tarsalis *mosquitoes. The majority of irrigation points (~71%) were in the western part of the state. Landesman et al. [24] demonstrated regional difference in precipitation associations with WNV disease incidence for the United States as a whole when split into east and west using the Mississippi River as a dividing line. They point out that this is likely due to the transition to *Cx. tarsalis *as the primary vector in the western United States. This is supported by the fact that the proportion of *Cx. tarsalis *positive mosquito pools increased as WNV moved westward in 2001–2004 [15]. Figure 7 demonstrates that in Iowa there is a transition as you move west to a greater proportion of *Cx. tarsalis *out of total *Culex *mosquitoes. Numerous studies have used degree days to explain or estimate the timing of mosquito populations and WNV disease occurrence [23,40,41], and this is an approach that might be used in Iowa in the future. The timing of vector abundances and WNV disease incidence needs to be studied in greater depth.

Other studies have demonstrated that climatic phenomena from the preceding year can be important for mosquito activity [42] and WNV disease incidence [24]. In Iowa, in 2003, 2004, and 2005 there were significantly lower mean precipitation values, annual average dewpoint temperatures, and annual average minimum temperatures in the preceding year for census block groups with WNV disease incidence. Similar to this study, Landesman et al. [24] found that 2003 and 2004 WNV disease incidence was associated with lower amounts of precipitation in the preceding year. They cite the theory put forth by Chase and Knight [42] that disruption of food-webs in preceding years by drought can lead to large outbreaks in wetland mosquitoes the next year. In Iowa, *Cx. tarsalis *populations were highest in 2004 [30], following drought in late summer 2003, but the number of human WNV disease cases and sentinel chicken seroconversions were lowest. In addition, no WNV positive mosquito pools were found in 2004 in Iowa. Similar to this study, Landesman et al. [24] also found that WNV disease incidence in 2002 was associated with greater precipitation in 2001. In Iowa, those census block groups with WNV disease incidence had greater precipitation totals in 2001 but not significantly so. These findings clearly indicate a need for more detailed temporal study of climatic effects.

## Conclusion

The dynamics of human WNV disease in Iowa show rural and agricultural association in Iowa with greater occurrence in the western part of the state. The rural nature of disease incidence, and association with agriculture in the state overall, suggests that *Cx. tarsalis *is the more important vector in the state. However, the inconsistency in the climatic associations over the five year study seem to indicate that there might not be consistent ecological dynamics leading to disease incidence over time and space. A future area of research should be detailed investigation of the temporal and spatial dynamics of entomological data in relation to WNV disease incidence. This research would be aided by the investigation of bird and mammal interactions with mosquito vectors. A more detailed temporal and spatial analysis of climatic and entomological data could begin to help untangle the complicated WNV disease dynamics at a more local scale in the northern Midwest.

## Competing interests

The authors declare that they have no competing interests.

## Authors' contributions

All authors were involved in conceiving the idea for the research. SB geocoded and aggregated the WNV disease incidence data to census block groups, provided interpretation of results, and contributed to the writing of the paper. JD was responsible for compiling, managing, and analyzing all of the landscape and climatic data and contributed to the writing of the paper. LB and BT provided entomological data, expertise on mosquitoes, and contributed to the writing of the paper. RS assisted in results interpretation and contributed to the writing of the paper.
